# Understanding the Effects of Colleague Participation and Public Cause Proximity on Employee Volunteering Intentions: The Moderating Role of Power Distance

**DOI:** 10.3389/fpsyg.2020.552867

**Published:** 2020-11-11

**Authors:** Jundong Hou, Ling Qian, Chi Zhang

**Affiliations:** ^1^School of Economics and Management, China University of Geosciences, Wuhan, China; ^2^School of Business Administration, Hubei University of Economics, Wuhan, China; ^3^Department of Marketing, University of Indianapolis, Indianapolis, IN, United States

**Keywords:** employee volunteering intentions, colleague participation, colleague position, public cause proximity, power distance

## Abstract

Many organizations encourage their employees to participate in charitable activities as part of their corporate social responsibility strategies. As a result, there has been an increased research interest in employee volunteering behavior. However, while previous research on employee volunteering decisions has focused on both individual-level and organizational-level factors, there has been less focus on peer involvement and volunteer cause proximity. To go some way to filling this research area, this paper conducted two studies to examine the possible effects of colleague participation, colleague position and public cause proximity on employee volunteering intentions. Study 1 found that colleague participation and public cause proximity had significant effects on employee volunteering, and Study 2 found that power distance played a moderating role in the relationship between colleague position and employee volunteering. This study contributes to theoretical research on employee volunteering and provides some information to assist firms retain engaged volunteers.

## Introduction

Organizations often encourage their employees to participate in charitable welfare activities in their local communities as part of their Corporate Social Responsibility (CSR) strategies (Basil et al., 2009). One of the most common ways that firms and employees try to “giveback” is through employee volunteering (Brockner et al., 2014). *Employee volunteering* has been identified as a classic “win-win-win” scenario (Caligiuri et al., 2013) as the firm enhances its reputation, employees upgrade their skills, and there are positive impacts for the charitable causes. Therefore, employee volunteering activity has become more common in many workplaces (Brockner et al., 2014), as well is often part of a company initiative (Rodell et al., 2016). For example, Rodell et al. (2016) estimated that more than 60% of companies had formal employee volunteering programs, and that about 90% of firms had some informal methods to encourage and support employee volunteering. Despite the growing interest in volunteering and the vital role that volunteers play in society, as corporate volunteering is still a relatively new CSR concept, it has limited magnitude (Dreesbach-Bundy and Scheck, 2017). Therefore, it is important to understand how employees can be motivated to participate in incorporating volunteering schemes, which are often on company time.

Generally, in-company volunteering initiatives are similar to social movements in that there is a collective effort aimed at addressing a broader social need (Muller et al., 2014). Because of the potential size of the volunteering workforce that could be generated by corporate volunteering schemes, collectively, companies have the potential to significantly influence action on national and global societal issues. For instance, in every community they serve, Darden Restaurant focuses on the battle against hunger (Rodell et al., 2017). In recent years, despite the increasing importance of employee volunteering in CSR practice, little is known about the nature of volunteer engagement and its correlates (Malinen and Harju, 2017). Accurately, academic research lacks understanding of whether regular employee volunteers really rely on or differentiate among the different incentive conditions. Even though is it not possible to gain an overall comprehensive picture of corporate volunteering in one study, the motivation in this paper is to extend existing conversations on volunteering by initiating a discussion of the situational factors from both co-worker and cause domains.

First, what is the colleague-specific driver that initiates corporate volunteering engagement? As part of corporate volunteering programs, companies generally provide resources to encourage and support employee volunteering; however, is this the best way to mobilize employee volunteering? Recent discussions on the peer effect have suggested that employees are influenced by the reference groups in which they operate, such as the colleagues around them. In fact, as a reference group, workplace colleagues often exert a greater influence on attitudes toward volunteering; however, the amount of influence a coworker has on another generally depends on how “close” the followers are to their colleagues (Howell et al., 2005). Therefore, a deeper understanding of how colleague engagement impacts employee volunteering intentions and actions could assist companies determine how to organize and mobilize employee volunteers to ensure sustainable long-term activities.

Second, what is the outside company trigger that activates employee volunteering intention? Research has found that individual volunteers benefit in terms of the improvements in the society. However, there are many causes such as health, education, or poverty, many of which have a certain distance between the volunteering campaign and the employees, which allows the employees to determine the personal costs such as the time and effort needed to volunteer. Accordingly, the volunteering activity traits that can affect followers and how these are evaluated by volunteering employees depend on the “distance” they are from the causes. Therefore, it is reasonable to infer that an employee’s choice to volunteer could be affected by public cause proximity. Although many studies have examined the effects of physical distance in corporate philanthropy (Schons et al., 2017) and individual donors (Zhu et al., 2017), few studies have sought to understand how employees react to the cause proximity associated with company volunteering activities. It is expected that a clearer understanding of this could assist in the development of corporate volunteering practices.

Additionally, business globalization means that many corporate volunteering campaigns are conducted in areas that have different cultural values. Therefore, it is necessary to explore how employee cultural values influence the understanding of colleague volunteering actions and cause proximity and the degree to which colleague engagement and cause proximity interact with followers’ cultural values to affect their volunteer behavior. Of four cultural value dimensions of Hofstede (2001), employee power distance was seen as the moderator for two reasons. First, power distance is one of the most important cultural values in most existing cultural value frameworks (Lin et al., 2013). Second, power distance is the most relevant cultural value factor in the current research framework, because employees with different cultural values may view organizational justice and supervisor support differently, and employees’ fundamental values regarding power are likely to affect their understanding of and their reaction to their supervisor’s volunteer behavior.

The motivation in this paper was based on the need to supplement the paucity of research, examining how peer involvement and cause proximity can impact employee volunteerism. Specifically, this paper conducted two studies, the first of which was to empirically investigate the main effects of colleague participation and cause proximity on employee volunteering intentions, and the second of which was to divide the work colleagues into two categories (e.g., peer and supervisor) to (1) examine the “crossover” effect between the position of a colleague involved in a volunteering activity and cause proximity on a follower’s volunteering decision and (2) determine whether individual employee views concerning power distance moderated the links between a colleague’s position and cause proximity on employee volunteering intentions.

This study potentially makes several contributions to the understanding of volunteering in the commercial world. First, previous studies have specifically taken organizational- and job-level engagement perspectives to examine whether perceived organizational support enhanced volunteer engagement and associated attitudes. However, as it remains unclear how the situational factors from both the work and cause domains interactively impact volunteer decisions, this paper responds to these calls. In particular, this paper provides evidence that the “crossover” process through which an employee volunteering action emerges is driven to some extent by a colleague-driven process led by their participative decisions (e.g., involvement or not) and positions (e.g., peer involvement or supervisor involvement) vs. cause-level proximity regarding the corporate volunteering program (e.g., local community or non-local). Second, while previous research has concluded that power distance is an important cultural value dimension and impacts the way individuals interpret and evaluates social information, the adaptive function associated with cultural power distance in multicultural environments has been widely ignored (Lin et al., 2013). This study, therefore, sought to identify the adaptive role of power distance by examining its enlarging effect on the relationships between colleague position and cause proximity and employee volunteering intentions. The results from this examination add to the thinking about reference groups and physical distances in the volunteering field and provide insights and guidance for enterprise volunteer projects.

## Literature Review and Hypotheses

As discussed above, *employee volunteering* is when the staff members in a company volunteer their time or skills to a nonprofit or charitable organization during a planned activity (Rodell et al., 2016). As is widely recognized, as volunteering fulfills a business’s social responsibilities, generates internal benefits, and also benefits the employees, it is often included in CSR strategies (Porter and Kramer, 2002; Cycyota et al., 2016; Cook and Burchell, 2018). In association with CSR, employee volunteering has been linked with positive perceptions, such as happiness (Rodell, 2013), well-being (Stukas et al., 2016), collective pride (Rodell et al., 2017), morale (Caligiuri et al., 2013), work attitudes (Brockner et al., 2014), job performance (Malinen and Harju, 2017), and company reputation and image (Jones et al., 2014), all of which highlight the importance of effective employee volunteering.

From a behavioral decision perspective, work motivation could be a useful framework for determining the traits that lead to a decision to volunteer (Pinder, 1998) as motivation is the attitude, intensity, and persistence of one’s efforts (Rodell et al., 2016). As volunteering is often seen as a specific effort or behavior, it is possible to apply the attitude, intensity, and persistence framework to measuring the motivation of employees to be involved in volunteering. Therefore, a *volunteering attitude* reflects an employee’s decision to devote some effort toward a volunteer activity rather than toward work, *volunteering intensity* denotes that how often the employee chooses to volunteer for the volunteer activity, and *volunteering persistence* refers to the time span an employee chooses to continue the volunteer activity; that is, the longevity of the volunteering activity. Rodell et al. (2016) state that researchers may use any of the three conceptualizations presented, whichever is most suitable depending on the study, thus our study focuses on the direction/attitude to volunteer.

### Antecedents for Employee Volunteering

The company-level and individual-level factors that influence employee volunteering have been identified in previous research, with the company-level factors including organizational support, job design, and work context. Organizational support was found to increase participation (Peterson, 2004; Malinen and Harju, 2017) and enhances volunteering intensity (Booth et al., 2009; Bowles, 2009). However, Kim and Kim (2016) identified multi-level company support determinants for employee volunteering that included individual, organizational, and institutional level factors. In addition to the multi-level organizational support factors, employee volunteering has also been found to be impacted by job characteristics. Currently, there are two different views as to how job design affects employee volunteer behavior. One approach is that if employees are full of passion and feel that their jobs are challenging, they will appreciate the organization by volunteering (Powell, 2006; Slattery et al., 2010). The other approach is that an employee’s participation in volunteering is because of compensatory motivations (Grant, 2012); that is, if employees feel their job performance lack significance, they seek to volunteer to obtain a sense of meaning (Edwards and Rothbard, 2000). Both these approaches have been examined in empirical studies (Pajo and Lee, 2011; Rodell, 2013).

Work context and volunteering climate can also influence employee volunteering. Some aspects of work context (i.e., work schedules, payment schedules, and job uncertainty) can be influential as they determine the level of the employee’s temporal and financial autonomy, which are crucial for planning and participating in volunteering activities (Rodell et al., 2016). Similarly, the corporate volunteering climate has the potential to influence all employees regardless of whether they take part in the volunteering programs because such a climate can have a meaningful effect on employee attitudes and behaviors through shared perceptions and experiences and encourage a positive, indirect relationship with affective commitment through collective pride (Rodell et al., 2017).

Researches on individual-level antecedents have documented the demographic, personality trait, motivational, and other psychological factors related to volunteering (Rodell et al., 2016; Ainsworth, 2020). Several studies have found that demographic factors such as gender, age (Cornwell and Warburton, 2014), family structure (Bandy and Ottoniwilhelm, 2012), education (Marshall and Taniguchi, 2012), and religious beliefs (Galen et al., 2015) impact employee involvement in social causes. Certain personality traits have also attracted considerable attention. The most common individual factors that have been associated with employee volunteering are being an extrovert (Finkelstein, 2009), having an individual risk propensity (Dong, 2015), and the big five traits (Erez et al., 2008). Psychological motivation has also been recognized as an important individual-level antecedent. Overall, however, both qualitative and quantitative investigations have found that employees who engage in volunteer programs are typically driven by a complex motivational mechanism (Kiviniemi et al., 2002; Peloza and Hassay, 2006; Stukas et al., 2016), which suggests that volunteering intentions may be related to multiple functions (Rodell et al., 2016) such as value shaping, understanding enhancement, protective, social and career functions (Stukas et al., 2016), and prosocial, social, and learning opportunity motivations (Hurst et al., 2019). Alongside these motivations, there may be other psychological factors that hold some impact over an employee’s volunteering decision, such as psychological pressure and psychological ownership (Ainsworth, 2020).

“Volunteer behavior is not dependent solely on the person or on the situation, but rather is dependent on the interaction of person-based dynamics and situational opportunities” (Clary et al., 1998). Based on this understanding, a comprehensive theoretical corporate volunteering framework is proposed to highlight how task, social, and knowledge characteristics affect sustained volunteering behavior (Grant, 2012). As previous empirical research has had conflicting findings regarding the impact of the multiple motivations on employee volunteering, it is important to concurrently consider the internal motivation and contextual factor effects. Several studies have concluded that psychological motivations and different social and situational factors could have different interactive influences on employee volunteering (Hu et al., 2016). However, despite the significant research into employee volunteering, the situational factors that enable or undermine the influence of internal motivation remain largely unclear (Finkelstein, 2009). Further, if personal and situational factors both uniquely contribute to employee volunteering, research on one factor alone would result in a narrow understanding of the corporate volunteering antecedents (Hu et al., 2016).

**Figure 1:**
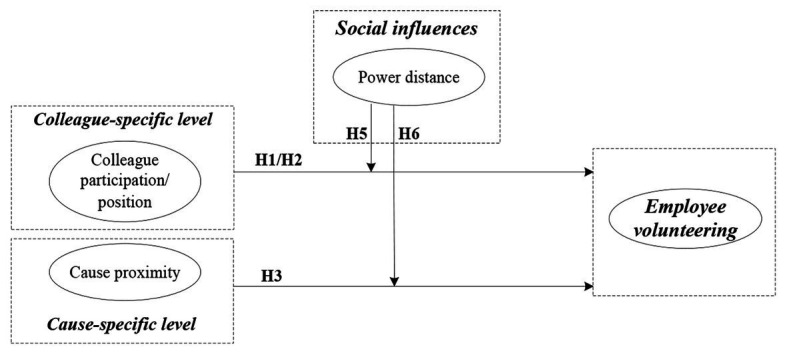
Conceptual model.

Based on the above discussion, the framework needs to include the source of the social contextual factors. While it is evident that social influences in the work domain, such as coworkers responses (e.g., superior and peer) to volunteer activities, can have normative effects on an employee’s decision to volunteer and possibly substitute for personal motives, public cause domain factors, such as the proximity to a specific social cause, are situational stimuli and reference points that can trigger and magnify the motivation to be engaged in the cause. Therefore, with its focus on the social influences and multiple levels of the work and cause domains, this paper attempts to offer a better understanding of the nature of employee volunteering (see Figure 1).

### Effect of Colleague Participation on Employee Volunteering

Volunteer campaign participation is any unpaid expenditure of time and energy outside work the company requires of its employees. Previous research has found that being engaged at work was positively correlated to employee performance (Malinen and Harju, 2017), and in a volunteering context, Huynh et al. (2014) found a negative relationship between work engagement and turnover intentions. However, the distinction between employee and colleague volunteer participation has yet to be revealed.

Social influence theory claims that people’s decisions are often intentionally or unintentionally affected by others (Cialdini and Goldstein, 2004). When making decisions, people often accept information from other people regardless of whether the information is correct or irrelevant (Rodell et al., 2016). However, people are more likely to accept advice from highly credible sources to comply with the wishes of others to achieve rewards or avoid punishments, to build close psychological associations, or to be accepted by the group, all of which means that people may change their decisions to conform with the reference group; an action that is often referred to as the peer effect or peer influence.

The amount of peer group pressure people perceive that they are under affects their decisions to perform or not to perform a behavior (White et al., 2009). Generally, as people tend to act within the frame of reference of the groups they belong to, reference groups can influence individual decision-making. Further, as members of a group, people tend to construct their identities based on relational or collective identification (Zhang et al., 2014), which means they may use social classification and internalized characteristics to classify themselves as belonging to a certain group. Symbolic interactionism means that a person’s actions or behaviors are based on the meanings that they attach to them (Blumer, 1969). If a society promotes volunteering, then volunteers may internalize related images of being a volunteer, which then influences their subsequent behavior. As local, more proximate units, employees often perceive workgroups to be cognitively closer than their companies (Mueller and Lawler, 1999) and, therefore, more strongly identify with their workgroup (Riketta and Dick, 2005).

Many studies have addressed why individuals want to affiliate with peers who have similar interests and the degree to which these peer relationships provide a rich context for the behavior socialization that could be considered misconduct (Choukasbradley et al., 2015). However, peer influence is not an inherently negative process. Previous studies in the United States, China, and the Netherlands found that peers can also lead/guide adolescents toward positive outcomes and prosocial behaviors (Masten et al., 2009; van Goethem et al., 2014; Choukasbradley et al., 2015; Law and Shek, 2015). Most past work on prosocial peer influence has been focused on adolescents, primarily because it has been surmised that at this age, relationships can be particularly influential. However, employees differ significantly from adolescents in terms of the potential prosocial behavior motivators.

Much of the related research on employee volunteering has examined the important role of corporate philanthropy for business purposes and volunteering’s potential to deliver tangible business benefits (Peloza et al., 2009); however, whether co-worker participation influences group volunteering intentions has rarely been examined. With some studies finding that employee behavior is influenced by a broad range of peers, such as employees’ colleagues or family members (Peloza et al., 2009). Further, if employees are part of a work culture that endorses particular social activities or volunteer participation, people could incur some costs if they choose to abstain (Peloza et al., 2009). Therefore, in this paper, it is surmised that employees are more willing to be actively involved in volunteering if their co-workers are taking part or have taken part in the past. Therefore, the following hypotheses are given:

*Hypothesis* 1: Colleague participation has a significant positive effect on employee volunteering.

In the workplace, according to the positions of colleagues involved into a volunteer campaign, they generally can be categorized as peer (e.g., colleagues with the same position) and superior (e.g., colleagues with a superior position). Therefore, we need further explore the effect of different positions of colleague on employee volunteering. It has been found that the effects of superior-launched and peer colleague-launched donation drives determined how much the employees donated (Du et al., 2014). Social identity theory claims that as part of their self-concept, individuals usually classify themselves into a certain group and internalize the group characteristics in the belief that this in-group perception facilitates greater love and reciprocal behavior than out-group members (Brewer, 1999). It was found that the knowledge of a shared group identity increased cooperative behavior, but the psychological mechanisms that underlie such in-group cooperation were less clear. Subsequently, Brewer (2008) examined general expectancy evidence on whether others were always cooperative within the in-group. Being given recognition and acceptance within the same social category has also been found to be sufficient for the formation of in-group preferences (Masson and Verkuyten, 2010). Compared to superiors such as managers and supervisors, peer colleagues of a similar ranking in the workplace are more easily accepted as in-group members, which indicates that the volunteering participation of peer colleagues could be a strong stimulus for other employees to mimic the behavior. Therefore, the following hypotheses are suggested:

*Hypothesis* 2: There is greater employee volunteering when a peer rather than a superior is involved in a volunteer campaign.

### Impact of Public Cause Proximity on Employee Volunteering

Enterprises mobilize or organize employees to participate in volunteering activities such as local community activities primarily or public causes, such as poverty alleviation or large-scale sports events. Cause proximity, which can be global, national, regional, or local, is the distance between the volunteering activities and the employees and is specifically related to employee or consumer reactions to CSR campaigns. Construal Level Theory, which is associated with psychological distance, claims that abstract and global features are more persuasive in products produced in spatially distant locations (such as a foreign country) than those produced in closer locations (such as a nearby town; Zhu et al., 2017). Applying this same logic to volunteering, Smith and Alcorn (1991) claimed that consumers were most concerned about local causes as these had a direct influence on their lives.

Geographic distance has been found to be a communication barrier for corporations as responses to social influences are often determined by immediacy or the proximity to physical sources (Landreth, 2002). Generally, consumers consider the impact of sources (such as other consumers) within a social space, with those located in the same social space being more influential. Therefore, geographic distance would be more likely to have a positive impact on volunteer intentions as employees would be more inclined to engage in volunteering activities that have a direct concrete or tangible influence on their lives (Zhu et al., 2017). Therefore, it is hypothesized that:

*Hypothesis* 3: Public cause proximity has a significant positive effect on employee volunteering;

The effects of the cues can be more important for one group than another when considering co-worker involvement in volunteer campaigns and public cause proximity. Specifically, co-worker pressure and public cause proximity cues could be more influential on those who are less involved in the promoted volunteer programs because these employees usually rely on peripheral reference points rather than directly evaluating the message arguments. Therefore, the influence of a superior rather than a peer and a local rather than a non-local impact could be seen as a cue to take part. These cues could be seen as reference messages that others associate with these employees, which in turn makes the volunteer campaign more desirable. While many employees may initially find the firm volunteer program less personally relevant, a superior’s participation can pressure the employee to become involved. Further, an emphasis on a proximal rather than a distant campaign could encourage greater employee involvement; therefore, colleague company position and public cause proximity could concurrently operate to influence employee volunteering intentions, which gives rise to the following hypothesis:

*Hypothesis* 4: Employee volunteering is greater when the volunteer program is targeted locally and involves a peer colleague rather than a superior.

### Moderating Effect of Power Distance

Power distance is the extent to which a less powerful individual expects and accepts unequally distributed power in institutions and organizations (Qian and Li, 2016). Employees who believe that supervisors should have a greater degree of authority over subordinates are considered to have high-power distance and vice versa (Wang and Guan, 2018). Therefore, power distance perceptions could have a significant great impact on employee participation and decision-making. In workplaces, it is typical for employees that have high power distance to accept status differences and as a subordinate, comply with their supervisor’s directives (Chen and Aryee, 2007). In groups with low power distance, however, employees feel less constrained by the expectations of the supervisor-subordinate relationship and are more likely to participate in the decision-making, express their ideas, and discuss policies with their supervisor because they believe they have equal rights. These employees also feel free to make their own choices without necessarily having to consider their superior’s opinions. Behavioral reinforcement of peer influence is a process through which employees obtain the social norms related to volunteering from their peer group, which in turn guides the employee’s decisions. In contrast, employee groups with high power distance are expected to accept the policies stipulated by the authority without question and obey the volunteer rules in the company. Therefore, even when some of their peers’ volunteer, all employees believe that it is appropriate to comply with the leader’s volunteering decisions; that is, the displayed behaviors are not reinforced. However, if the superior is involved in volunteering, the employees may perceive greater value and insights from their supervisor’s authoritative mentorship and feedback (Qian et al., 2018). Therefore, it is surmised that both employees with low and high levels of power distance would tend to value their supervisor’s opinion and may even be inclined to see the volunteering campaign as mandatory because of this hierarchical and authoritative pressure. Therefore, the following hypothesis is proposed:

*Hypothesis* 5: Employee volunteering is greater when the power distance is lower for peer colleagues. However, there are no distinctions when a superior is involved.

As discussed, employees who have low power distance are often empowered to engage in and discuss volunteering decisions and are less motivated to volunteer because of their supervisory mentors. As they may also initially find the community volunteering campaign less personally relevant, an emphasis on the proximal local community benefits rather than the distant community benefits of the volunteering services may shed new light as to the relevancy of that activity. This is because the volunteering is directed toward the local community and employees who physically share the same space.

People in high power distance groups, however, have a greater psychological dependence on their supervisors for clear goals and specific actions (Cole et al., 2013). Therefore, when making a decision to volunteer, as employees with high power distance rely more on their supervisor’s directions, whether the activity is physically closer to the volunteer may be less important. Therefore, the following hypothesis is proposed:

*Hypothesis* 6: Power distance moderates the positive relationship between cause proximity and employee volunteering in such a way that the relationship is stronger for employees with lower rather than higher power distance.

## Preliminary Survey

This research conducted two studies to reliably test the proposed conceptual framework. Study 1 explored the effects of colleague participation and public cause proximity on employee volunteering. After the influence of colleague participation on employee volunteering was confirmed and as it was not clear whether the involved colleague’s position impacted other employees’ volunteering decisions, and Study 2 was conducted to reveal the effects of colleague position and public cause proximity on volunteering and the moderating role of power distance.

**Figure 2:**
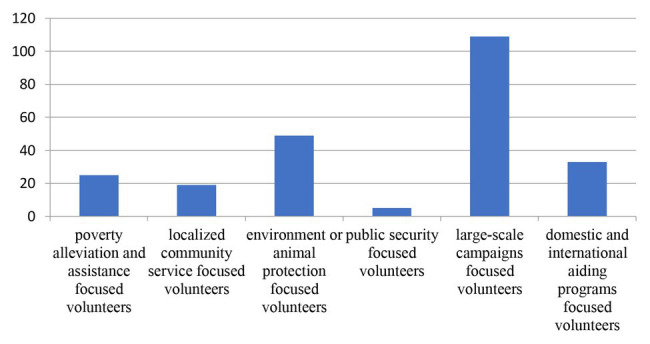
Frequencies of the six volunteer campaign types.

A preliminary study was conducted to determine the employees’ most preferred volunteering activity as the stimulus materials in both studies. The volunteering programs were first sorted into six categories based on Tian (2007); poverty alleviation and assistance focused volunteers, localized community service focused volunteers, environmental or animal protection focused volunteers, public security focused volunteers, large-scale campaign focused volunteers, and domestic and international aid program focused volunteers. Then, the employees were asked to choose one to three of the volunteer projects in which they would be most likely to participate. Participants were recruited through a professional survey agency (Sojump.com) in exchange for a small payment. A total of 103 responses (61 female, *M*_age_ = 26.29, ranging from 22 to 35 years, 42.60% of respondents coming from private company) were collected, with the results showing that large-scale campaign focused volunteering was the most highly ranked, as shown in Figure 2.

Therefore, this was used as a reference for the situational stimulus in Study 1. As there are many types of large-scale campaigns, this study focused on large-scale sports events, such as marathons, because these events are heavily dependent on volunteers and have age, gender, professional experience, and socio-economic status limitations. Further, large-scale sports events generally have well-established management processes and organizational systems to allow for a mix of experienced and new volunteers. Finally, large-scale sports events are usually held in many different communities of a city or all over the country, making it easier to assess the cause proximity.

To enhance the external validity of our research, environmental protection was chosen for the stimulus material in Study 2, which was ranked second in the preliminary survey.

## Study 1

### Design and Method

The hypotheses were tested using a two (yes vs. no) colleague participation and a two (near versus distant) public cause proximity one-factor between-subjects design; therefore, there were four possible scenarios as shown in Table 1.

**Table 1:** Scenarios in Study 1.

Conditions		Description in the survey
S1	Colleague participation (yes)	You have learnt that your colleague is going to sign up for this sport event
S2	Colleague participation (no)	You have learnt that your colleague is not going to sign up for this sport event
S3	Public cause proximity (high)	You have learnt that the volunteering programs in this sport event were made to a local community near your company’s location
S4	Public cause proximity (low)	You have learnt that the volunteering programs in this sport event were made to a non-local community far away from your company’s location

To manipulate cause proximity, the participants were told that the volunteering programs were made to a local community near the company’s location or a non-local community far away from its location. A total of 320 MBA students were recruited from two comprehensive universities in Wuhan, China in 2017 in exchange for wining a gift voucher, all of whom had been full-time employees in Chinese enterprises for more than 3 years. The participants were randomly assigned to the colleague participation and cause proximity conditions, and they were presented with a brief description of the volunteer programs for a specific large-scale sport event, as follows.

Because of the large-scale and tight schedules and the many athletes, referees, journalists, and audiences associated with modern sports events, there are significant challenges and a greater number of staff are needed. Generally, large-scale volunteer recruitment for service design, operations, and sports activity management is needed. Recently, you heard from your co-worker in the same company that an international sports event is to be held and is seeking volunteers.

After reading the description, the participants were asked to keep their current volunteering intentions in mind while answering the questions to capture the measures associated with their volunteering. To assess employee volunteering, participants completed a four-item, 7-point scale (1 = strongly disagree, 7 = strongly agree) adapted from Rodell (2013): “I am willing to give my time to this sports event”; “I am willing to apply my skills in ways that benefit this sports event”; “I am willing to devote my energy toward this sports event”; and “I am willing to employ my talents to aid this sports event.” A total of 293 completed this study (i.e., S1/S2: *n* = 161 and S3/S4: *n* = 132). The participants ranged in age from 25 to 42, with an average age of 31.56 (*SD* = 5.23), and approximately 58% were male. The coefficient *α* for this scale is 0.941.

### Hypotheses Tests

It was predicted that the employee volunteering intention would differ between the yes and no questions about whether the co-worker volunteered (Hypothesis 1) in local or non-local community volunteering programs (Hypothesis 3). To assess these predictions, a binary split was first conducted on the colleague participation measure to identify the yes and no colleague involvement and on the cause proximity measure to assess the employees’ low or high proximity to a community volunteer program. Then, one-way ANOVA analyses were performed on the dependent volunteering measures to determine the direct effects.

Hypothesis 1 predicted that there would be a greater motivation to volunteer by others if a co-worker were involved. The one-way ANOVA results indicated that there was a significant direct effect for colleague involvement in volunteering (*M*_S1_ = 5.103, *SD* = 1.460; *M*_S2_ = 4.278, *SD* = 1.486; *F* = 6.970, *p* < 0.001). For the Hypothesis 3 public cause proximity prediction, the one-way ANOVA indicated that there was a significant difference (*F* = 3.776, *p* < 0.001) for local (*M*_S3_ = 4.871, *SD* = 1.275) and non-local (*M*_S4_ = 4.308, *SD* = 1.314) volunteering, which indicated that locally directed volunteer programs would enhance employee attitudes toward the volunteering activity; therefore, Hypothesis 3 was supported.

### Discussion

The results of Study 1 generally supported the predictions. As expected, the main effects of colleague involvement and cause proximity demonstrated that followers that were more involved with the cause tended to be more interested in participating to help the cause, and that local rather than non-local volunteer campaigns incited more favorable volunteering intentions, which also suggested that these employees were willing to consider volunteer campaigns even if they were not personally relevant. This possible finding could be useful to volunteer program developers, as it suggests that focusing on local issues could be the key to arousing the interests of employees in volunteering. Although Study 1 has determined the main effect of colleague participation on employee volunteering, it has not directly explained whether the position of involved colleague affects their volunteering decision, nor verified the specific role of power distance in this effect. Moreover, the selected volunteering campaign was focused on large-scale sports event in Study 1, while actually volunteer activities cover a wide range of as shown in Figure 2, thus whether the effects observed in Study 1 are robust in these fields remains to be explored. Therefore, Study 2 will focus on these two aspects above.

## Study 2

### Design and Method

Study 1 revealed the main effects for colleague participation and public cause proximity on employee volunteering motivations. However, it was not clear whether the positions of the colleagues engaged in the volunteer programs encouraged other employees to volunteer. To investigate these different effects on the three employee volunteering dimensions and to assess the moderating effect of power distance, a two colleague position (peer vs. superior) × two public cause proximity (low vs. high) between-subjects factorial design was conducted in Study 2, for which there were also four scenarios, as shown in Table 2. In this study, participants were presented with a brief description of the volunteer programs for a specific environmental protection event.

**Table 2:** Scenarios in Study 2.

Conditions		Description in the survey
S5	Colleague position (peer) + public cause proximity (high)	You have learnt that your peer colleague is going to sign up for the environmental protection activity near the company location
S5	Colleague position (peer) + public cause proximity (low)	You have learnt that your peer colleague is going to sign up for the environmental protection activity far from the company’s location
S7	Colleague position (superior) + public cause proximity(high)	You have learnt that your superior is going to sign up for a volunteer program regarding an environmental protection activity near the company location
S8	Colleague position (superior) + public cause proximity(low)	You have learnt that your superior is going to sign up for a volunteer program regarding an environmental protection activity far from the company’s location

Requests to participate in the research were emailed through a volunteering organization in Wuhan, Hubei Province, China in 2018, after which surveys were firstly distributed to 20 human resource managers from different firms, who in turn were asked to distribute our invitation to (around 15) their colleagues. Each manager randomly assigned their participants to one of the four Study 2 conditions and could anonymously and voluntarily complete the questionnaire online or call the researchers to have a hard copy sent.

In addition to evaluate volunteering intention with the same instruments as in Study 1 (*α* = 0.877), power distance was also measured by asking participants to report their perceptions of power distance in the workplace using a five-item measure adapted from Dorfman and Howell (1988). Sample items on this scale were “Superiors should make most decisions without consulting subordinates” and “Employees should not disagree with management decisions” (1 = strongly disagree, 7 = strongly agree), and with higher scores indicating that the employees had higher power distance orientations. The coefficient alpha for the power distance measure was 0.853.

Three hundred questionnaires were distributed and 193 were completed (a 64.3% response rate), where, S5: *n* = 52, S6: *n* = 49, S7: *n* = 43, and S8: *n* = 48. On average, the respondent employees were 27.28 years old (*SD* = 3.14) two-thirds male (66.3%), with a bachelor’s degree (57.0%). Around 39.9% were from private enterprises and 29.0% were from state-owned enterprises, and 67.9% had been involved in volunteering.

### Hypotheses Tests

In line with Hypothesis 2, to understand the relationship between the colleague’s position and volunteering intention, an ANOVA analysis was conducted with colleague position as the independent variable and volunteering intention as the dependent variables. It was found that there was a significant difference in the volunteering (*F* = 24.591, *p* < 0.001, *η*^2^ = 0.115), with increased volunteering intentions demonstrated when the colleague was a peer (*M*_peer_ = 4.885, *SD* = 1.168) than when the colleague was a superior (*M*_superior_ = 4.059, *SD* = 1.205), which supported Hypothesis 2.

To test Hypothesis 4, a 2 × 2 full-factorial ANOVA was performed on volunteering intentions. The results indicated significant main effects for colleague position (*F* = 24.591, *p* < 0.001, *η*^2^ = 0.115) and public cause proximity (*F* = 10.132, *p* = 0.002, *η*^2^ = 0.051); however, the interaction effect between colleague position and public cause proximity (*F* = 1.891, *p* = 0.171, *η*^2^ = 0.010) did not reach the *p* < 0.05 level of significance.

**Figure 3:**
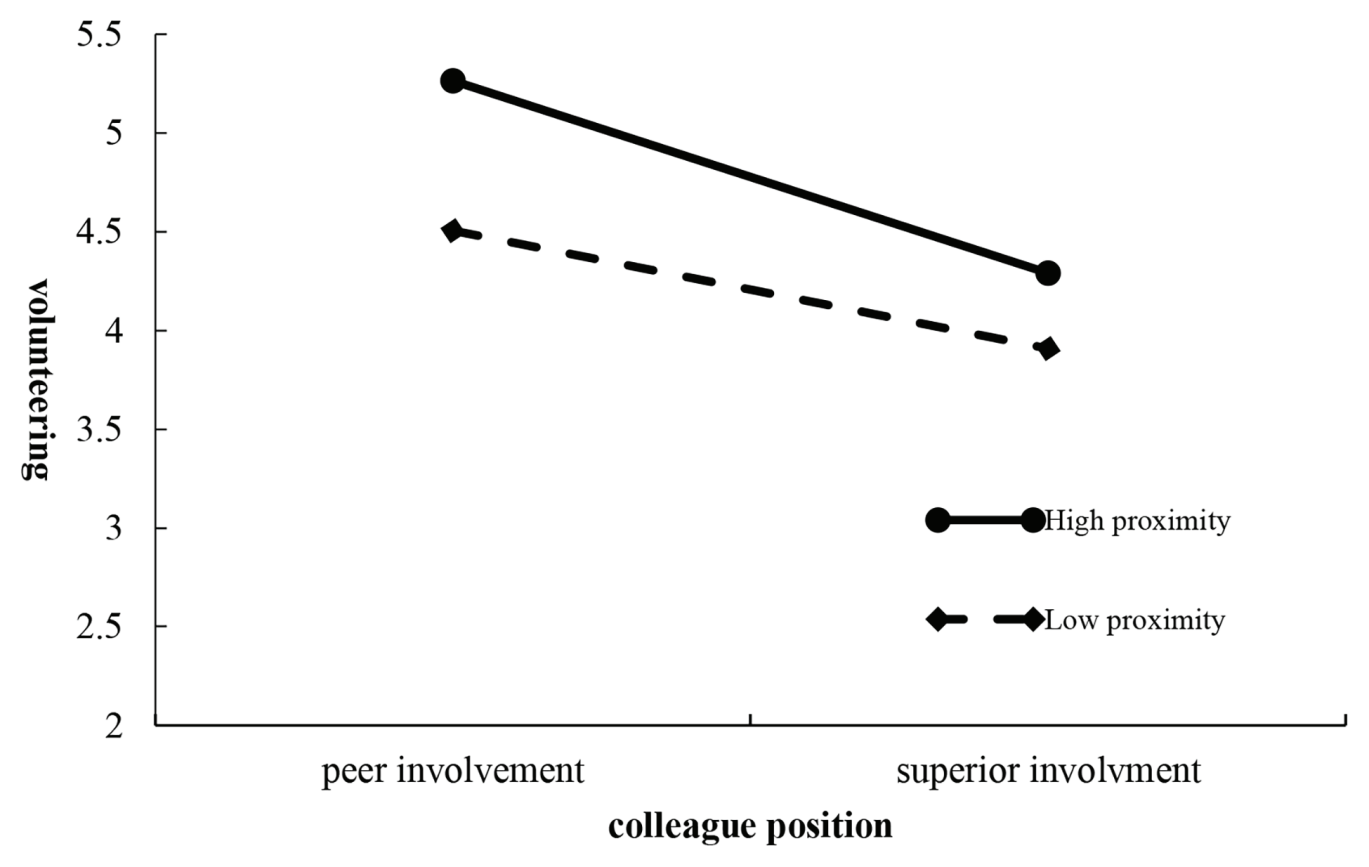
Colleague position and public cause proximity on volunteering.

Next, a comparison of the mean values was necessary to accurately identify the predicted effects in Hypothesis 4. The first cell of interest for this hypothesis was for peer involved employees across the two cause proximity levels. For the first volunteering intentions comparison, the analysis assessed whether the involved peers responded more favorably when the volunteer program was nearer to the company’s location (*M* = 5.264, *SD* = 1.038) rather than far away (*M* = 4.505, *SD* = 1.181) and found the predicted significant difference (*F* = 10.917, *p* = 0.001, *η*^2^ = 0.055); Figure 3 shows the two plots for means. Interestingly, superiors involved with the cause were not expected to engender more favorable behavior when employees were directed toward local rather than non-local volunteer campaigns, which was consistent with the findings (*M*_High proximity_ = 4.209, *SD* = 1.199; *M*_Low proximity_ = 3.908, *SD* = 1.204; *F* = 1.559, *p* = 0.213, *η*^2^ = 0.008).

More specifically, while it was posited that the peers involved in a cause would be more likely to respond favorably to volunteer campaigns near the company locations, no significant differences were posited for volunteer activities that involved superiors between the high and low proximity respondents. In sum, these comparisons to assess the veracity of the Hypothesis 4 predictions did indicate that it was important, where volunteer programs were targeted. Although public cause proximity was not found to influence the employee evaluations of their superiors’ involvement, it remains important for their peer colleagues.

**Figure 4:**
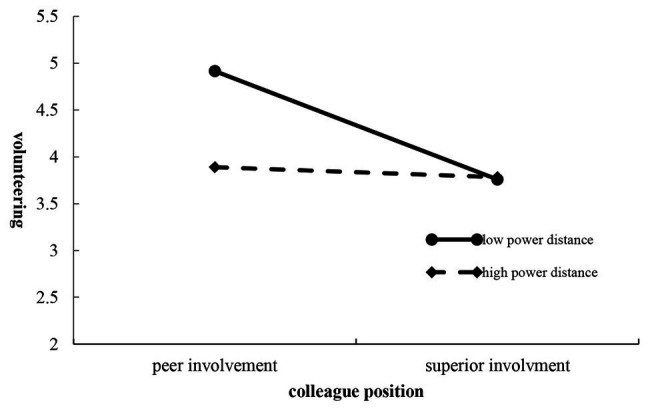
Colleague position and power distance on volunteering.

Hypothesis 5 predicted that power distance played a moderating role in the effect of colleague position on employee volunteering. The data were split into a high and a low power distance group based on the median. The overall 2 × 2 ANOVA showed a significant main effect for colleague position (*F* = 8.716, *p* = 0.004, *η*^2^ = 0.044), indicating that employees that had a peer involved in the cause viewed volunteering campaigns more favorably (*M* = 4.583) than those who had a superior involved (*M* = 4.028), which again supported Hypothesis 2. In the assessment of the significant main effect of power distance, it was found that employees with low power distance (*M* = 4.537,) would be more likely to volunteer than those with high power distance (*M* = 4.075, *F* = 6.038, *p* = 0.015, *η*^2^ = 0.031). The univariate results showed that there was a significant interaction effect for colleague position and power distance in volunteering (*F* = 10.497, *p* = 0.001, *η*^2^ = 0.053). Further analysis revealed that, it was found that power distance significantly impacted volunteering intentions when a peer was involved in the volunteer campaign (*M*_Low power distance_ = 5.119, *SD* = 1.075; *M*_High power distance_ = 4.048, *SD* = 1.139, *F* = 14.492, *p* < 0.001, *η*^2^ = 0.071), which suggested that if a peer colleague were involved, the volunteering intentions would be greater in employees with low rather than high power distance (Figure 4 shows the two plots for the means). However, no such differences were found for when a superior was involved (*M*_Low power distance_ = 3.955, *SD* = 0.938; *M*_High power distance_ = 4.102, *SD* = 1.335, *F* = 10.348, *p* = 0.556, *η*^2^ = 0.002); therefore, this specific comparison offered some support for Hypothesis 5.

To test Hypothesis 6, a two public cause proximity × two power distance full-factorial ANOVA was performed on volunteering intention, the results yielded a significant main effect for public cause proximity (*F* = 9.286, *p* = 0.003, *η*^2^ = 0.047) and power distance *F* = 14.074, *p* < 0.001, *η*^2^ = 0.069); however, the interaction effect between public cause proximity and power distance (*F* = 0.014, *p* = 0.905, *η*^2^ = 0) was not significant at *p* < 0.05, which supported Hypothesis 3 but did not provide enough evidence to support Hypothesis 6.

We used the bootstrapping approach in order to confirm the moderating effect of power distance (Hayes, 2017), running our analysis using 5,000 bootstraps for Model 1. The results show that the interaction of colleague position and power distance is significant (*b* = 1.218, *t* = 3.240, *p* = 0.001, 95% CI: 0.477–1.960). However, we found significance of conditional effect of colleague position on employee volunteering at low levels of power distance (Effect = −0.415, Boot LLCI = −1.633, and Boot ULCI = −0.696) but not at high levels (Effect = 0.585, Boot LLCI = −0.521, and Boot ULCI = 0.629), providing support for Hypothesis 5. We also found no support for Hypothesis 6 since moderation is not evident (*b* = 0.042, *t* = 0.119, *p* = 0.905, 95% CI: −0.646 to 0.729), meaning that the indirect effect of public cause proximity on employee volunteering intentions does not hold for both low and high levels of power distance.

### Discussion

The primary goals of Study 2 were to determine whether volunteering was dependent on a colleague’s position, to assess the interaction effect for colleague participation and public cause proximity, and to determine the moderating effect of power distance. The findings indicated that there were more favorable attitudes toward volunteering if a peer colleague were involved. In particular, there was a more positive reaction when a peer coworker engaged in a local volunteer campaign rather than a non-local campaign; however, no such difference was found for the involvement of their superiors. Employees with different power distances were found to have different responses to high or low proximity volunteer programs. Taken as a whole, the results from Study 2 suggested that the employees were not completely resistant to persuasion and could benefit from volunteering campaign cues that addressed attitudes, intensity, and persistence.

## General Discussion and Implications

The overarching goal of these two studies was to shed light on the interactive impact of the situational work and cause domain factors on employee volunteering. Results of this study found that coworker engagement positively affected the volunteering behavioral intentions of peer employees rather than superiors. These conclusions were consistent with social influence theory, which implies that individual behavior is influenced by the internalized moral principles of reference groups. From a social identity approach, employees make the same decision as in-group members to maintain their consistency within the group. This peer-to-peer effect results from the attitudinal and behavioral traits of a psychologically relevant reference group rather than from perceived pressure from a superior. These results supported the argument that social factors are important for workplace volunteering contexts and also provide suggestions as to the type of variables that should be targeted in interventions designed to encourage employee volunteering engagement. Therefore, when a company is seeking to implement a CSR strategy using employee volunteering, it needs to mobilize other personnel (and especially peers) to ensure a more positive response.

Public cause proximity was also tested to be regarded as an effective cue for volunteer recruitment in the workplace. Generally, when employees take part in a volunteer program, they usually focus on the local rather than the non-local community. Research on public cause attributes have mainly focused on cause-related marketing. Landreth (2002) proposed three attributes (importance, proximity, and consistency) for public causes and demonstrated the significance of public cause proximity to consumer behavior marketing. In this study, it was found that employees were more inclined to volunteer if the campaign were local, possibly because people view the local community as more salient regardless of the importance of the target volunteer activity. This conclusion was in line with the findings of good cause marketing and pro-social behavior.

We further examine an important boundary condition of employee power distance in order to fully understand the context in which the link holds (i.e., coworker participation-employee volunteering intentions). In line with the hypothesized effect, results demonstrate that power distance significantly impacted volunteering intentions when a peer was involved in the volunteer campaign, while no such differences were found for when a superior was involved. The conditional effect of coworker participation on volunteering intentions is significant for low power distance employees.

### Theoretical Contributions

We provide two contributions to the related research fields at least. First, previous research on influential employee volunteering factors have mainly focused on individual and organizational factors and tended to neglect the importance of the roles of reference groups and physical distance in shaping employee volunteer behavior. This study explored how the situational factors from both the work and cause domains interactively impact volunteer decisions, which further provides evidence that the “crossover” process through which an employee volunteering action emerges is driven to some extent by a colleague-driven process led by their participative decisions and positions vs. cause-level proximity regarding the corporate volunteering program. This can contribute to optimize the corporate volunteering campaigns based on the features of their target audiences. Second, our research contributes to employees’ dispositions within the cultural values literature (e.g., power distance). Power distance has been studied in a variety of contexts, such as organizational behavior and team management, and has tended to focus on the effect of power distance on interpersonal relationships. To the best of our knowledge, however, there has been little research that has examined the effect of power distance on volunteering or on the role of power distance in maintaining group norms. We challenge and extend findings that the influence of a coworker engagement on volunteering behavioral intentions of employees with different levels of power distance could be different.

### Practical Implications

These results have some implications for firms involved in designing volunteer campaigns. First, we provide a clear applicability to situational strategy on employee volunteering programs. Our results suggest that both coworker participation and public cause proximity could be effective cues for volunteer recruitment in the workplace. Therefore, this research advances the collective knowledge regarding how to effectively trigger employee’s engagement when executing corporate volunteering campaigns. When enterprises are seeking to organize employee volunteering in their local communities, they could gain competitive advantage. This does not mean that the companies should only focus on local volunteer programs and neglect national or global campaigns; rather, it means that firms could focus on local angles for national causes and concentrate on local volunteering for national or international activities. Another possibility would be to ally with a general volunteer program and then develop a partnership with both national and local volunteer associations. For example, if a firm chose to partner with a community organization fighting poverty, it could support the Chinese Young Volunteers Association at a national level by demonstrating support for industrial development and management training and could also support a local community by providing related technologies and experiences for local residents who want to develop certain businesses to alleviate poverty. This tactic could allow firms to develop a volunteer program on multiple proximity levels.

Moreover, this study found that power distance acted as a moderator in the relationship between colleague position and employee volunteering and that employees with lower power distance were less receptive to inequalities. It was also found that employees were more willing to participate in volunteering with their peer colleagues than with their superiors and that coworker volunteer engagement was an important predictor for behavioral intention. Therefore, to increase employee volunteering, volunteer program visibility could be increased so that employees know which colleagues are already engaging in the target volunteering cause. Taken together, all these results should provide some clues to assist marketers in answering important strategic issues related to corporate volunteering when implementing CSR strategy.

## Limitations and Directions for Future Research

Although this research has theoretical contributions and offers some practical implications, there are several limitations that need to be addressed in future studies. First, the research focused exclusively on outcomes relevant to volunteering to examine the interactive effects of colleague participation and public cause proximity. Although we tested the hypotheses using two studies, the findings were limited as only two types of stimuli were explored. It is possible that employees would respond differently depending on what the volunteer program entailed and who benefited. To address this gap, in future research, scholars need to employ more stimuli to further confirm their generalizability and improve external validity. Second, generally, volunteering motivation has been described in terms of the direction, intensity, and persistence of effort; however, the cross-sectional design of this study made it difficult to determine whether volunteering direction could be substituted with attitude. Therefore, in future research, a longitudinal research design or experimental design should be used to examine this issue. Third, the current study was also limited as it only sought to explain the moderating role of power distance at the individual level; therefore, it is still unclear as to whether this type of volunteering culture has a similar moderating effect at the national level. Further testing using cross-cultural research is needed to examine this moderating effect. Fourth, this research only assessed behavioral intentions under an imagined participation with a volunteer campaign as opposed to actual participation; that is, actual involvement with a volunteer campaign might produce different results. This weakness is common for studies in this field because attitudes and intentions toward social issues tend to be artificially high; therefore, future research needs to seek to gain a better understanding of signal usage by measuring actual volunteering behavior in the workplace.

## Conclusion

As employee volunteering programs are effective strategies for corporate social responsibility, many companies have been making greater efforts and dedicating greater resources to volunteer campaigns. However, there has a paucity of research on how company employees actually respond to these types of campaigns. The present study contributes to the theory of employee volunteering by revealing that coworker power distance orientations help explain employee reactions to volunteering. These findings suggest that focusing on colleague participation and public cause proximity could lead to a better understanding of the impact on employee volunteering intentions. In addition, the interaction of colleague position and power distance in relation to employee volunteering is a potentially interesting field that requires further examination.

## Data Availability Statement

The raw data supporting the conclusions of this article will be made available by the authors, without undue reservation.

## Ethics Statement

The study involving human participants were reviewed and approved by the ethics committee of China University of Geosciences. Written informed consent was inferred through the completion of the survey.

## Author Contributions

JH analyzed and interpreted the data and prepared the draft. LQ helped to recollect data and wrote the draft. CZ reviewed the draft critically and improved the important contents. All authors contributed to the article and approved the submitted version.

### Conflict of Interest

The authors declare that the research was conducted in the absence of any commercial or financial relationships that could be construed as a potential conflict of interest.

## References

[ref1] Ainsworth J. (2020). Feelings of ownership and volunteering: examining psychological ownership as a volunteering motivation for nonprofit service organisations. J. Retail. Consum. Serv. 52:101931. 10.1016/j.jretconser.2019.101931

[ref2] Bandy R. Ottoniwilhelm M. (2012). Family structure and income during the stages of childhood and subsequent prosocial behavior in young adulthood. J. Adolesc. 35, 1023–1034. 10.1016/j.adolescence.2012.02.010, PMID: 22414561 PMC3432915

[ref3] Basil D. Z. Runte M. S. Easwaramoorthy M. Barr C. (2009). Company support for employee volunteering: a national survey of companies in Canada. J. Bus. Ethics 85, 387–398. 10.1007/s10551-008-9741-0

[ref4] Blumer H. (1969). Symbolic interactionism: Perspective and method. Englewood Cliffs, NJ: Prentice Hall.

[ref5] Booth J. E. Park K. W. Glomb T. M. (2009). Employer-supported volunteering benefits: gift exchange among employers, employees, and volunteer organizations. Hum. Resour. Manag. 48, 227–249. 10.1002/hrm.20277

[ref6] Bowles M. P. (2009). Corporate social responsibility as support for employee volunteers: impacts, gender puzzles and policy implications in Canada. J. Bus. Ethics 84, 405–416. 10.1007/s10551-008-9716-1

[ref7] Brewer M. B. (1999). The psychology of prejudice: in-group love and out-group hate? J. Soc. Issues 55, 429–444.

[ref8] Brewer M. B. (2008). “Depersonalized trust and ingroup cooperation” in Modern pioneers in psychological science: An APS-Psychology Press series. Rationality and social responsibility: Essays in honor of Robyn Mason Dawes. ed. Krueger J. I. (New York, US: Psychology Press), 215–232.

[ref9] Brockner J. Senior D. Welch W. (2014). Corporate volunteerism, the experience of self-integrity, and organizational commitment: evidence from the field. Soc. Justice Res. 27, 1–23. 10.1007/s11211-014-0204-8

[ref10] Caligiuri P. Mencin A. Jiang K. (2013). Win-win-win: the influence of company sponsored volunteerism programs on employees, NGOs and business units. Pers. Psychol. 66, 825–860. 10.1111/peps.12019

[ref11] Chen Z. X. Aryee S. (2007). Delegation and employee work outcomes: an examination of the cultural context of mediating processes in China. Acad. Manag. J. 50, 226–238. 10.5465/amj.2007.24162389

[ref12] Choukasbradley S. Giletta M. Cohen G. L. Prinstein M. J. (2015). Peer influence, peer status, and prosocial behavior: an experimental investigation of peer socialization of adolescents’ intentions to volunteer. J. Youth Adolesc. 44, 2197–2210. 10.1007/s10964-015-0373-2, PMID: 26525387 PMC5985442

[ref13] Cialdini R. B. Goldstein N. J. (2004). Social influence: compliance and conformity. Annu. Rev. Psychol. 55, 591–621. 10.1146/annurev.psych.55.090902.142015, PMID: 14744228

[ref14] Clary E. G. Snyder M. Ridge R. D. Copeland J. Stukas A. A. Haugen J. (1998). Understanding and assessing the motivations of volunteers: a functional approach. J. Pers. Soc. Psychol. 74, 1516–1530. 9654757 10.1037//0022-3514.74.6.1516

[ref15] Cole M. S. Carter M. Z. Zhang Z. (2013). Leader-team congruence in power distance values and team effectiveness: the mediating role of procedural justice climate. J. Appl. Psychol. 98, 962–973. 10.1037/a0034269, PMID: 24060159

[ref16] Cook J. Burchell J. (2018). Bridging the gaps in employee volunteering: why the third sector doesn’t always win. Nonprofit Volunt. Sect. Q. 47, 165–184. 10.1177/0899764017734649

[ref17] Cornwell B. Warburton E. (2014). Work schedules and community ties. Work. Occup. 41, 139–174. 10.1177/0730888413498399

[ref18] Cycyota C. S. Ferrante C. J. Schroeder J. M. (2016). Corporate social responsibility and employee volunteerism: what do the best companies do? Bus. Horiz. 59, 321–329. 10.1016/j.bushor.2016.01.004

[ref19] Dong H. K. (2015). The effects of individual risk propensity on volunteering. Nonprofit Manag. Leadersh. 26, 5–18. 10.1002/nml.21139

[ref20] Dorfman P. W. Howell J. P. (1988). Dimensions of national culture and effective leadership in patterns. Adv. Int. Comp. Manag. 3, 127–150.

[ref21] Dreesbach-Bundy S. Scheck B. (2017). Corporate volunteering: a bibliometric analysis from 1990 to 2015. Bus. Ethics Eur. Rev. 26, 240–256. 10.1111/beer.12148

[ref22] Du L. Zhao F. Zhang C. (2014). Impact of mobilization context on employees’ donation intentions in China. Soc. Behav. Personal. 42, 115–124. 10.2224/sbp.2014.42.1.115

[ref23] Edwards J. R. Rothbard N. P. (2000). Mechanisms linking work and family: clarifying the relationship between work and family constructs. Acad. Manag. Rev. 25, 178–199. 10.5465/amr.2000.2791609

[ref24] Erez A. Mikulincer M. van Ijzendoorn M. H. Kroonenberg P. M. (2008). Attachment, personality, and volunteering: placing volunteerism in an attachment-theoretical framework. Personal. Individ. Differ. 44, 64–74. 10.1016/j.paid.2007.07.021

[ref25] Finkelstein M. A. (2009). Intrinsic vs. extrinsic motivational orientations and the volunteer process. Personal. Individ. Differ. 46, 653–658. 10.1016/j.paid.2009.01.010

[ref26] Galen L. W. Sharp M. Mcnulty A. (2015). Nonreligious group factors versus religious belief in the prediction of prosociality. Soc. Indic. Res. 122, 411–432. 10.1007/s11205-014-0700-0

[ref27] Grant A. M. (2012). Giving time, time after time: work design and sustained employee participation in corporate volunteering. Acad. Manag. Rev. 37, 589–615. 10.5465/amr.2010.0280

[ref28] Hayes A. F. (2017). Introduction to mediation, moderation, and conditional process analysis: A regression-based approach. NY: Guilford Publications.

[ref29] Hofstede G. (2001). Culture’s consequences: Comparing values, behaviors, institutions, and organizations across nations. 2nd Edn. Thousand Oaks, CA: Sage.

[ref30] Howell J. M. Neufeld D. J. Avolio B. J. (2005). Examining the relationship of leadership and physical distance with business unit performance. Leadersh. Q. 16, 273–285. 10.1016/j.leaqua.2005.01.004

[ref31] Hu J. Jiang K. Mo S. Chen H. Shi J. (2016). The motivational antecedents and performance consequences of corporate volunteering: when do employees volunteer and when does volunteering help versus harm work performance? Organ. Behav. Hum. Decis. Process. 137, 99–111. 10.1016/j.obhdp.2016.08.005

[ref32] Hurst A. Coyne E. Kellett U. Needham J. (2019). Volunteers motivations and involvement in dementia care in hospitals, aged care and resident homes: an integrative review. Geriatr. Nurs. 40, 478–486. 10.1016/j.gerinurse.2019.03.010 30922706

[ref34] Huynh J. Y. Xanthopoulou D. Winefield A. H. (2014). The job demands-resources model in emergency service volunteers: examining the mediating roles of exhaustion, work engagement and organizational connectedness. Work Stress 28, 305–322. 10.1080/02678373.2014.936922

[ref35] Jones D. A. Willness C. R. Madey S. (2014). Why are job seekers attracted by corporate social performance? Experimental and field tests of three signal-based mechanisms. Acad. Manag. J. 57, 383–404. 10.5465/amj.2011.0848

[ref36] Kim J. Kim T. (2016). Multi-level antecedents of company support for employee volunteering. Corp. Soc. Responsib. Environ. Manag. 23, 37–49. 10.1002/csr.1360

[ref37] Kiviniemi M. T. Snyder M. Omoto A. M. (2002). Too many of a good thing? The effects of multiple motivations on stress, cost, fulfillment, and satisfaction. Personal. Soc. Psychol. Bull. 28, 732–743. 10.1177/0146167202289003

[ref38] Landreth S. (2002). For a good cause: The effects of cause importance, cause proximity, congruency and participation effort in consumers’ evaluations of cause related marketing. Detroit: Proquest Company Press.

[ref39] Law B. M. F. Shek D. T. L. (2015). Beliefs about volunteerism, volunteering intention, volunteering behavior, and purpose in life among Chinese adolescents in Hong Kong. Sci. World J. 9, 855–865. 10.1100/tsw.2009.32 PMC5823204 19734959

[ref40] Lin W. Lei W. Chen S. (2013). Abusive supervision and employee well-being: the moderating effect of power distance orientation. Appl. Psychol. 62, 308–329. 10.1111/j.1464-0597.2012.00520.x

[ref41] Malinen S. Harju L. (2017). Volunteer engagement: exploring the distinction between job and organizational engagement. Voluntas 28, 69–89. 10.1007/s11266-016-9823-z

[ref42] Marshall G. A. Taniguchi H. (2012). Good jobs, good deeds: the gender-specific influences of job characteristics on volunteering. Voluntas 23, 213–235. 10.1007/s11266-011-9188-2

[ref43] Masson C. N. Verkuyten M. (2010). Prejudice, ethnic identity, contact and ethnic group preferences among dutch young adolescents. J. Appl. Soc. Psychol. 23, 156–168. 10.1111/j.1559-1816.1993.tb01058.x

[ref44] Masten C. L. Juvonen J. Spatzier A. (2009). Relative importance of parents and peers: differences in academic and social behaviors at three grade levels spanning late childhood and early adolescence. J. Early Adolesc. 29, 773–799. 10.1177/0272431608325504

[ref45] Mueller C. W. Lawler E. J. (1999). Commitment to nested organizational units: some basic principles and preliminary findings. Soc. Psychol. Q. 62, 325–346.

[ref46] Muller A. R. Pfarrer M. D. Little L. M. (2014). A theory of collective empathy in corporate philanthropy decisions. Acad. Manag. Rev. 39, 1–21. 10.5465/amr.2012.0031

[ref47] Pajo K. Lee L. (2011). Corporate-sponsored volunteering: a work design perspective. J. Bus. Ethics 99, 467–482. 10.1007/s10551-010-0665-0

[ref48] Peloza J. Hassay D. N. (2006). Intra-organizational volunteerism: good soldiers, good deeds and good politics. J. Bus. Ethics 64, 357–379. 10.1007/s10551-005-5496-z

[ref49] Peloza J. Hudson S. Hassay D. N. (2009). The marketing of employee volunteerism. J. Bus. Ethics 85, 371–386. 10.1007/s10551-008-9734-z

[ref50] Peterson D. K. (2004). Recruitment strategies for encouraging participation in corporate volunteer programs. J. Bus. Ethics 49, 371–386. 10.1023/b:busi.0000020872.10513.f2

[ref51] Pinder C. C. (1998). Work motivation in organizational behavior. Upper Saddle River, NJ: Prentice Hall.

[ref52] Porter M. E. Kramer M. R. (2002). The competitive advantage of corporate philanthropy. Harv. Bus. Rev. 80, 56–68. PMID: 12510538

[ref53] Powell G. G. N. (2006). When work and family are allies: a theory of work-family enrichment. Acad. Manag. Rev. 31, 72–92. 10.5465/amr.2006.19379625

[ref54] Qian J. Li X. (2016). Supervisory mentoring and employee feedback seeking: the moderating effects of power distance and political skill. Curr. Psychol. 35, 486–494. 10.1007/s12144-015-9317-y

[ref55] Qian J. Li X. Song B. Wang B. Wang M. Chang S.. (2018). Leaders’ expressed humility and followers’ feedback seeking: the mediating effects of perceived image cost and moderating effects of power distance orientation. Front. Psychol. 9:563. 10.3389/fpsyg.2018.00563, PMID: 29720956 PMC5915548

[ref56] Riketta M. Dick R. V. (2005). Foci of attachment in organizations: a meta-analytic comparison of the strength and correlates of workgroup versus organizational identification and commitment. J. Vocat. Behav. 67, 490–510. 10.1016/j.jvb.2004.06.001

[ref57] Rodell J. B. (2013). Finding meaning through volunteering: why do employees volunteer and what does it mean for their jobs? Acad. Manag. J. 56, 1274–1294. 10.5465/amj.2012.0611

[ref58] Rodell J. B. Booth J. E. Lynch J. Zipay K. (2017). Corporate volunteering climate: mobilizing employee passion for societal causes and inspiring future charitable action. Acad. Manag. J. 60, 1662–1681. 10.5465/amj.2015.0726

[ref59] Rodell J. B. Breitsohl H. Schroder M. Keating D. J. (2016). Employee volunteering: a review and framework for future research. J. Manag. 42, 55–84. 10.1177/0149206315614374

[ref60] Schons L. M. Cadogan J. Tsakona R. (2017). Should charity begin at home? An empirical investigation of consumers’ responses to companies’ varying geographic allocations of donation budgets. J. Bus. Ethics 144, 559–576. 10.1007/s10551-015-2832-9

[ref61] Slattery J. P. Selvarajan T. T. Anderson J. E. Sardessai R. (2010). Relationship between job characteristics and attitudes: a study of temporary employees. J. Appl. Soc. Psychol. 40, 1539–1565. 10.1111/j.1559-1816.2010.00628.x

[ref62] Smith S. M. Alcorn D. S. (1991). Cause marketing: a new direction in the marketing of corporate responsibility. J. Serv. Mark. 5, 21–37.

[ref63] Stukas A. Hoye R. Nicholson M. Brown K. Aisbett L. (2016). Motivations to volunteer and their associations with volunteers’ well-being. Nonprofit Volunt. Sect. Q. 45, 112–132. 10.1177/0899764014561122

[ref64] Tian J. (2007). Voluntary behavior theory and practice. Shanghai: Lixin Accounting Press.

[ref65] van Goethem A. J. van Hoof A. van Aken M. G. de Castro B. Raaijmakers Q. W. (2014). Socialising adolescent volunteering: how important are parents and friends? Age dependent effects of parents and friends on adolescents’ volunteering behaviours. J. Appl. Dev. Psychol. 35, 94–101. 10.1016/j.appdev.2013.12.003

[ref66] Wang H. Guan B. (2018). The positive effect of authoritarian leadership on employee performance: the moderating role of power distance. Front. Psychol. 9:357. 10.3389/fpsyg.2018.00357, PMID: 29628902 PMC5876282

[ref67] White K. M. Smith J. R. Terry D. J. Greenslade J. H. Mckimmie B. M. (2009). Social influence in the theory of planned behaviour: the role of descriptive, injunctive, and in-group norms. Br. J. Soc. Psychol. 48, 135–158. 10.1348/014466608X295207, PMID: 18435863

[ref68] Zhang S. Chen G. Chen X. P. Liu D. Johnson M. D. (2014). Relational versus collective identification within workgroups. J. Manag. 40, 1700–1731. 10.1177/0149206312439421

[ref69] Zhu L. Yi H. Chen Q. Miao H. (2017). It’s the thought that counts: the effects of construal level priming and donation proximity on consumer response to donation framing. J. Bus. Res. 76, 44–51. 10.1016/j.jbusres.2017.03.007

